# Multi-Band MIMO Antenna Design with User-Impact Investigation for 4G and 5G Mobile Terminals

**DOI:** 10.3390/s19030456

**Published:** 2019-01-23

**Authors:** Naser Ojaroudi Parchin, Haleh Jahanbakhsh Basherlou, Yasir I. A. Al-Yasir, Atta Ullah, Raed A. Abd-Alhameed, James M. Noras

**Affiliations:** 1 Faculty of Engineering and Informatics, School of Electrical Engineering and Computer Science, University of Bradford, Bradford BD7 1DP, UK; Y.I.A.Al-Yasir@bradford.ac.uk (Y.I.A.A.-Y.); A.Ullah5@bradford.ac.uk (A.U.); R.A.A.Abd@bradford.ac.uk (R.A.A.-A.); J.M.Noras@bradford.ac.uk (J.M.N.); 2 Microwave Technology Company, Ardabil 56158-46984, Iran; Hale.Jahanbakhsh@gmail.com

**Keywords:** 4G, 5G, double-element slot antenna, LTE, MIMO system, mobile terminal, user-impact

## Abstract

In this study, we propose a design of a multi-band slot antenna array applicable for fourth-generation (4G) and fifth-generation (5G) smartphones. The design is composed of double-element square-ring slot radiators fed by microstrip-line structures for easy integration with radio frequency (RF)/microwave circuitry. The slot radiators are located on the corners of the smartphone printed circuit board (PCB) with an overall dimension of 75 × 150 mm^2^. The proposed multiple-input multiple-output (MIMO) antenna is designed to meet the requirements of 4G and 5G mobile terminals with essential bandwidth for higher data rate applications. For −10 dB impedance bandwidth, each single-element of the proposed MIMO design can cover the frequency ranges of 2.5–2.7 GHz (long-term evolution (LTE) 2600), 3.45–3.8 GHz (LTE bands 42/43), and 5.00–5.45 GHz (LTE band 46). However, for −6 dB impedance bandwidth, the radiation elements cover the frequency ranges of 2.45–2.82 GHz, 3.35–4.00 GHz, and 4.93–5.73 GHz. By employing the microstrip feed lines at the four different sides of smartphone PCB, the isolation of the radiators has been enhanced and shows better than 17 dB isolation levels over all operational bands. The MIMO antenna is implemented on an FR-4 dielectric and provides good properties including S-parameters, efficiency, and radiation pattern coverage. The performance of the antenna is validated by measurements of the prototype. The simulation results for user-hand/user-head impacts and specific absorption rate (SAR) levels of the antenna are discussed, and good results are achieved. In addition, the antenna elements have the potential to be used as 8-element/dual-polarized resonators.

## 1. Introduction

With the development of wireless communication techniques, multiple-input multiple-output (MIMO) technology is attracting attention [1]. It is known that MIMO operation can highly improve the data rate, capacity, and link reliability of wireless communication systems [2]. MIMO technology is currently used in fourth-generation (4G) user equipment with the frequency band of 2.6 GHz and is going to be used in the future fifth-generation (5G) portable devices [3]. The 3.6 GHz frequency band appears promising for sub-6 GHz MIMO 5G communications [4].

MIMO antennas are key elements in modern wireless communication systems because they can minimize interference without increased bandwidth. Recently, several designs of MIMO antennas were reported for future smartphone applications [5,6,7,8,9,10,11]. However, all these antennas only support one mobile terminal, and they have some limitations, such as being narrow-band. To the best of the authors’ knowledge, there are only a few MIMO designs covering both 4G and 5G spectrums [12,13,14,15]. In contrast to the reported designs, each radiator of our design can cover 4G and 5G frequency operation bands simultaneously, which makes the proposed MIMO antenna more suitable for future smartphones.

The slot antenna has been investigated extensively for different wireless systems because of its attractive features including being light-weight, its compactness, and it ease of integration with radio frequency (RF) circuits [16,17]. Its multiband characteristics make it the most suitable choice for multi-mode communications in the modern wireless systems [18,19].

We propose here a new multi-band MIMO antenna design providing wide impedance-bandwidth and improved isolation properties for three different operation bands of the current and future mobile terminals. The antenna contains four double-element square-ring slot radiators placed at different corners of the smartphone printed circuit board (PCB) to provide full radiation coverage. The proposed system exhibits good characteristics in terms of the fundamental radiation properties. In addition, its performance in the presence of the user-hand and user-head has been studied.

This paper is organized as follows: Section 2 brings out the geometry and characteristics of the single-element multi-band slot antenna design. Simulation and measured results of the proposed 4G/5G MIMO antenna is presented in Section 3. In Section 4, the characteristics of the smartphone antenna in the vicinity of the user have been studied. Section 5 concludes this study.

## 2. Double-Element Square-Ring Slot Antenna

**Figure 1:**
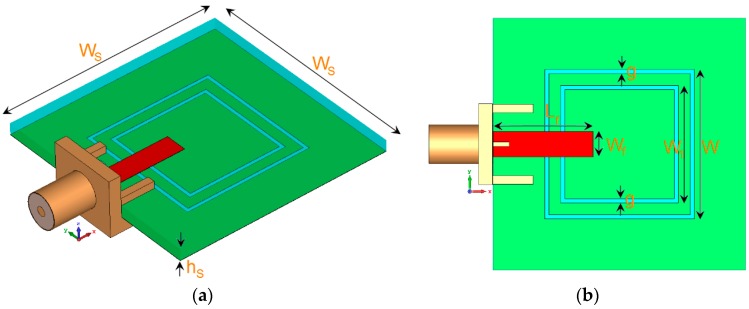
(**a**) Side and (**b**) top views of the proposed triple-band antenna.

The characteristics of the double-element square-ring slot resonator with various design parameters are investigated in this section. The configuration of the proposed single-element design is shown in Figure 1. The antenna is printed on an FR-4 substrate whose relative permittivity, loss tangent, and thickness are 4.4, 0.025, and h_S_ = 1.6 mm, respectively. For this antenna, it contains a pair of square-ring slots etched in the ground plane and a 50 Ω rectangular microstrip feed-line. The gap between the employed slot-ring is 0.3 mm. The parameter values for the single-element and also the proposed MIMO array are listed in Table 1.

**Figure 2:**
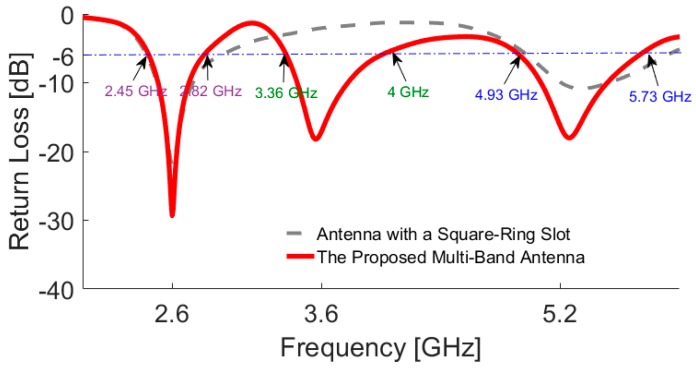
Simulated return losses for the antenna with single and double-element square-ring slots.

The motive behind the presented design is to achieve a compact multi-band antenna which can be integrated with a smartphone PCB. The return loss of the antenna with single and double-element square-ring slots is illustrated in Figure 2. The resonant frequencies of the antenna are mainly determined by the circumference length of the employed square-ring slots. The first resonance (at 2.6 GHz for the low-band) is mainly determined by the circumference of the outer square-ring slot. The second resonance (at 3.6 GHz for the middle-band) depends on the inner annular ring. Therefore, the circumference length of the square-ring slot needs to satisfy the dielectric wavelength at the corresponding frequency point, where W/2 + g = λ_1_ and W_1_/2 + g = λ_2_. However, the length of feed-line (L_f_) also has a little effect on the frequency point. The third resonance (at 5.25 GHz for the upper-band) is the second harmonic of the outer square-ring. It should be also noted that the second harmonic of the inner square-ring occurs above 7 GHz and is not discussed in this study.

**Figure 3:**
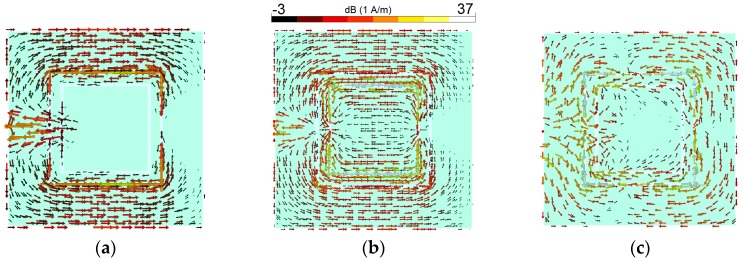
Current distributions at (**a**) 2.6 GHz, (**b**) 3.6 GHz, and (**c**) 5.25 GHz.

In order to further understand the triple-band characteristic, simulated surface current densities at 2.6, 3.6, and 5.25 GHz are shown in Figure 3. The maximum scale for each plot is the same. As seen in Figure 3a, the current flow reverses on the interior edge of the first resonator (2.6 GHz). The second resonator (second square-ring slot) has high current density and appears very active at 3.6 GHz, as shown in Figure 3b. At 5.25 GHz, Figure 3c shows that the exterior of the first resonator is more active, and most of the current is concentrated around it since it is the second harmonic of the first resonator [20]. However, there is always some coupling between the employed square-ring slots, and the frequency response of the antenna is a result of these complex interactions.

**Figure 4:**
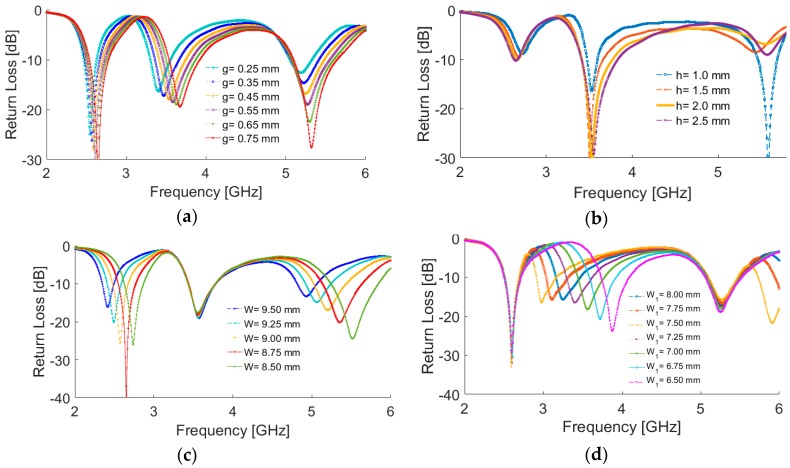
Return loss results for different values of (**a**) g (square-slot width), (**b**) h (substrate thickness), (**c**) W (the size of the first square-ring resonator), and (**d**) W_1_ (the size of the second square-ring resonator).

The results of varying fundamental design parameters g, h, W, and W_1_ are shown in Figure 4a–d. Figure 4a shows the effects of g (square-slot width) on the impedance matching of the antenna: the operational frequency bands of the antenna can be easily tuned by changing the value of g. The antenna return loss for different values of h (substrate thickness) is presented in Figure 4b, which shows that the isolation characteristic of the antenna at different frequencies is highly dependent on the thickness of the substrate. Figure 4c shows that W (the size of the first square-ring resonator) has a significant impact on the first and third resonant frequencies and very little impact on the second resonant frequency. The antenna return loss results for different values of W_1_ (the size of the second square-ring resonator) are plotted in Figure 4d. As evident from the figure, unlike W, it has very little impact on the first and third resonant frequencies. However, the second resonant frequency is influenced and tuned significantly. Based on the obtained results from Figure 4c,d, we can conclude that the operational frequency of the first channel (2.6 GHz of 4G) and the second channel (3.6 GHz of 5G) can be modified without affecting each other, which gives an independent-frequency-tuning characteristic useful for practical applications.

**Figure 5:**
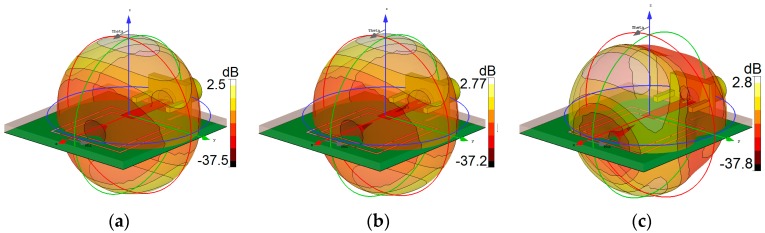
3D transparent views of the radiation patterns at (**a**) 2.6 GHz, (**b**) 3.6 GHz, and (**c**) 5.25 GHz.

**Figure 6:**
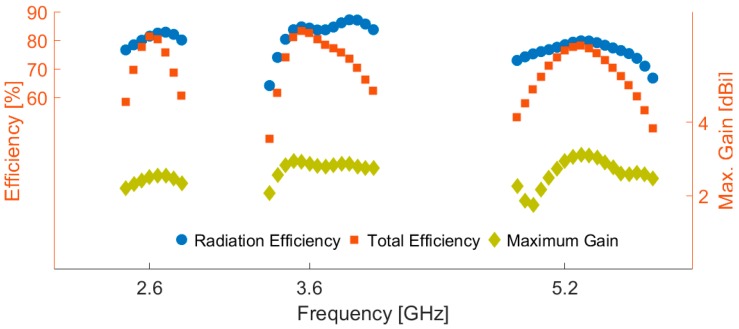
Maximum gain and efficiencies of the triple-band slot antenna.

The radiation patterns of the antenna at resonant frequencies (2.6, 3.6, and 5.25 GHz) are displayed in Figure 5: almost identical radiation performance with dumbbell-shaped patterns and 2.5–2.8 dB realized gain values are obtained at the selected frequencies. Note that the realized gain is the power gain of the antenna including mismatch losses. Maximum gain and efficiencies of the antenna over the operation bands are shown in Figure 6. More than 80% radiation and total efficiencies with more than 3 dBi maximum gains have been achieved at the resonant frequencies. Generally, the antenna provides high efficiencies with sufficient maximum gain levels, even though it has been implemented on a high-loss FR-4 dielectric. In the design, the insertion of the double square-ring slots appears to generate a discontinuity in the ground plane, which causes the electric current launched by the primary radiator to reroute its path along the conducting surface of the ground, thereby increasing the electrical length of the ground plane. With the strong coupling from the radiator, the ground slots have a considerable impact on the input impedance. This positive coupling effect is responsible for increasing the gain and efficiency characteristics of the antenna [21].

**Figure 7:**
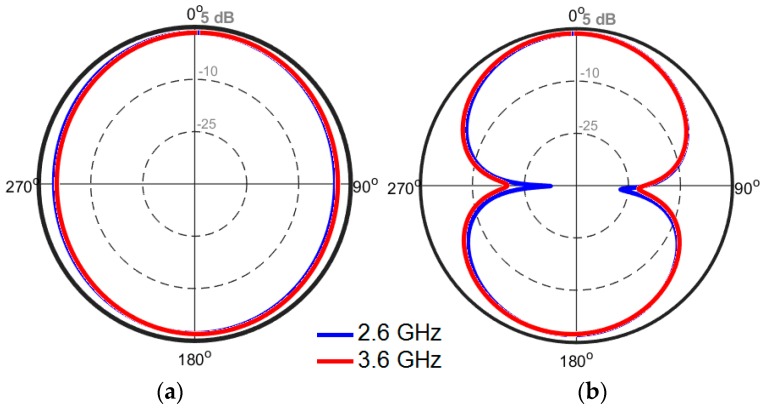
2D-polar radiation patterns for (**a**) H-plane and (**b**) E-plane.

**Figure 8:**
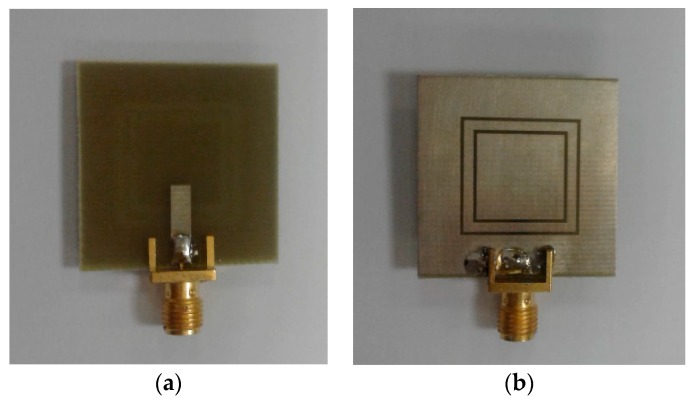
Fabricated antenna, (**a**) top view and (**b**) bottom view.

**Figure 9:**
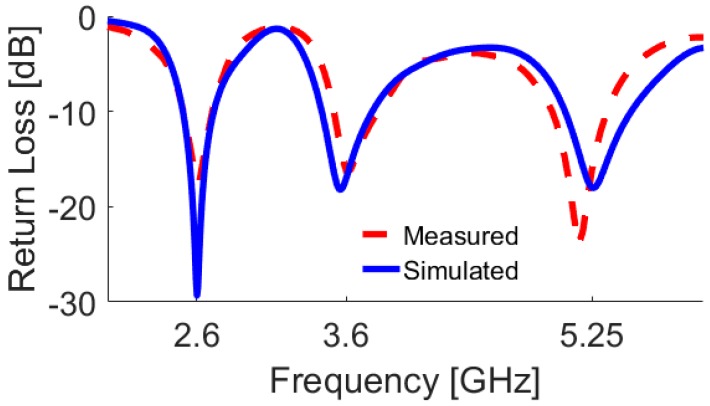
Measured and simulated return losses of the multi-band double-element slot antenna.

The 2D-polar radiation patterns of the antenna at 2.6 GHz and 3.6 GHz are illustrated in Figure 7. The antenna provides a nearly omnidirectional radiation patterns in the H-plane, while an 8-shaped radiation pattern in the E-plane has been achieved for different resonant frequencies. To verify the obtained results from the simulations, a prototype was fabricated and tested. Figure 8a,b shows the top and bottom views of the prototype, respectively. The simulated and measured results of the antenna return loss are illustrated in Figure 9. As seen, with a −10 dB impedance bandwidth, the antenna can cover the frequency bands of 2.5–2.7 GHz, 3.45–3.8 GHz, and 5–5.45 GHz with better than −18 dB return loss over all bands. Furthermore, there is good agreement between simulation and measurement.

## 3. The Proposed Multi-Band Smartphone Antenna

**Figure 10:**
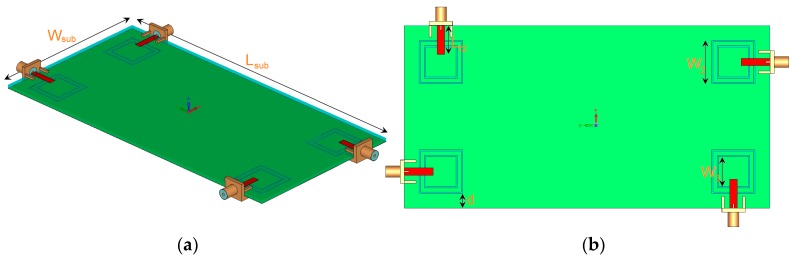
(**a**) Side and (**b**) top views of the antenna.

**Figure 11:**
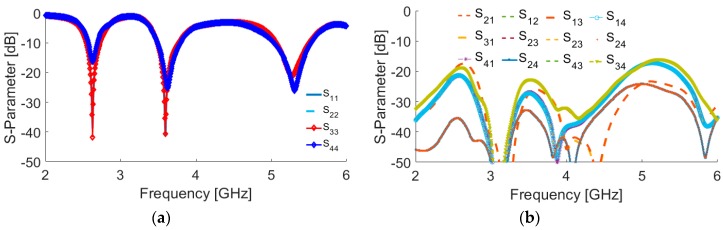
Simulated S-parameters (**a**) S_nn_ (input-impedance) and (**b**) S_nm_ (maximum mutual coupling).

The antenna is illustrated in Figure 10. It is constructed on a commercially available FR-4 dielectric substrate with a dimension of 75 × 150 × 1.6 mm^3^. As illustrated, four elements of the proposed triple-band double-element square-ring slot radiators are placed at the four corners of the smartphone PCB. Since each radiation element of the proposed MIMO system can work separately as transmitter and receiver, it is not necessary to include the feeding network. However, for phased array designs such as millimeter-wave 5G antennas, the feeding network should be included in the final design [22]. The simulated S-parameters are shown in Figure 11: the radiation elements of the antenna provide similar performance with high impedance matching at 2.6, 3.6, and 5.25 GHz. In addition, the maximum mutual coupling is less than −17 dB.

Employing the microstrip feed lines at four different sides of the PCB not only exhibits sufficient bandwidth, but also provides almost symmetrical radiation patterns to provide full radiation coverage. Moreover, it improves the isolation characteristic and decreases the mutual coupling between the antenna radiators.

**Figure 12:**
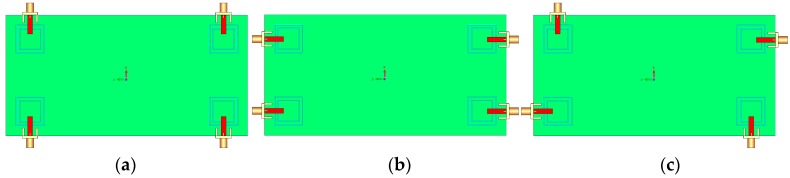
Different placement of the employed rectangular microstrip feed lines. (**a**) first, (**b**) second, and (**c**) third (the proposed).

**Figure 13:**
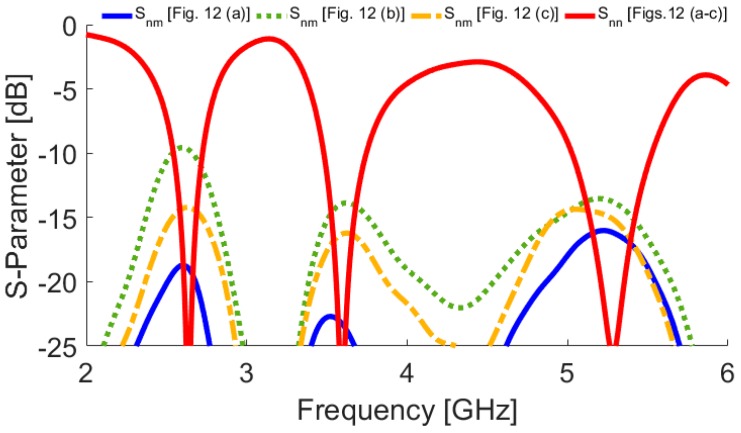
S_nn_ and S_nm_ characteristics of the antenna for different placements of the feed lines.

**Figure 14:**
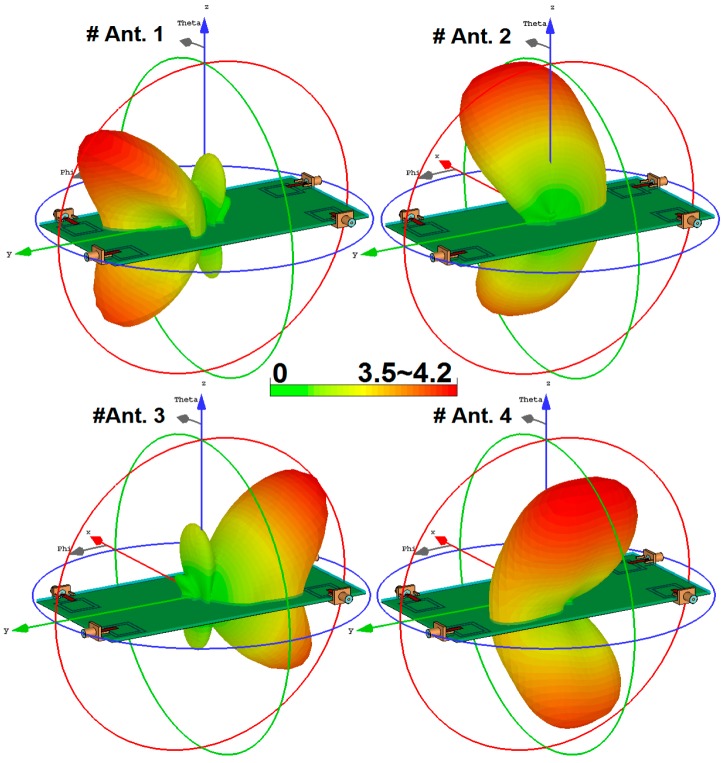
3D linear-scaling radiation patterns of the different elements at 3.6 GHz.

The configuration of the antenna with different placements of the microstrip feed lines are shown in Figure 12. Figure 13 depicts their S_nn_ (input-impedance) and S_nm_ (maximum mutual coupling) characteristics. It is evident that with these placements of its feed lines, the proposed design exhibits the lowest mutual coupling of less than −19, −23, and −17 dB coupling at 2.6, 3.6, and 5.25 GHz, respectively. The radiation pattern of the antenna elements (with linear scaling) at the middle frequency (3.6 GHz) is shown in Figure 14. It is clear that the proposed design (Figure 12c) can provide good radiation coverage on different sides of the PCB, while this cannot be obtained using the other feed-line placements (Figure 12a,b).

**Figure 15:**
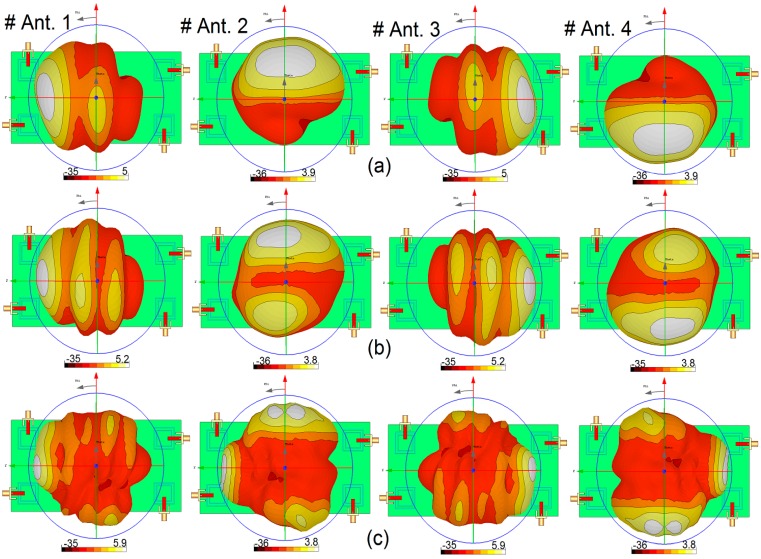
3D radiation patterns at (**a**) 2.6 GHz, (**b**) 3.6 GHz, and (**c**) 5.25 GHz.

**Figure 16:**
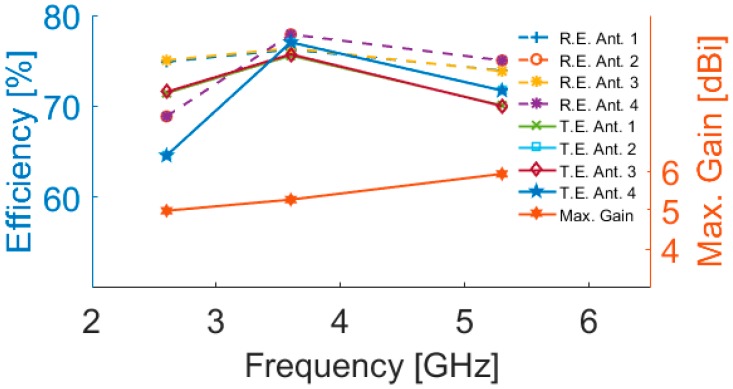
The antenna maximum gain and efficiencies.

3D radiation patterns for each antenna element at different resonant frequencies are illustrated in Figure 15: each side of the smartphone PCB can be covered by the radiators with sufficient gain values. The antenna maximum gain and its radiation efficiency (R.E.) and total efficiency (T.E.) for the different radiators are illustrated in Figure 16. As can be seen, more than 75% and 65% radiation and total efficiencies are obtained for the elements at the resonant frequencies. The antenna also exhibits around 5–6 dBi maximum gain values at 2.6, 3.6, and 5.25 GHz.

**Figure 17:**
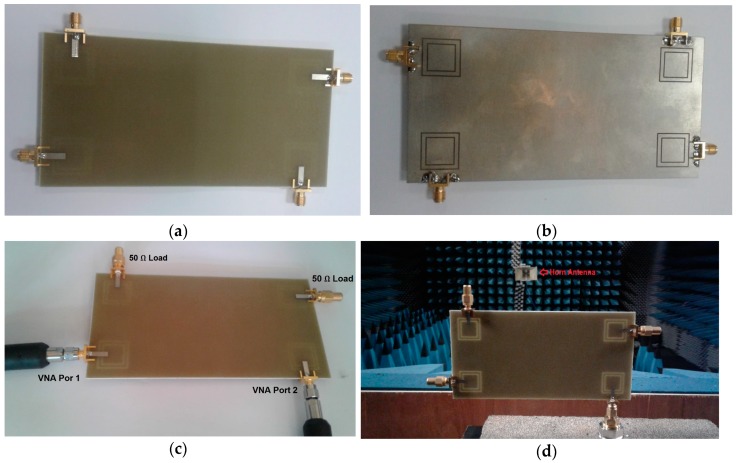
(**a**) Top and (**b**) bottom views of the prototype; measurements setups of (**c**) S-parameters and (**d**) radiation patterns.

The proposed MIMO slot antenna design was fabricated, and its characteristics, including the S-parameters and 2D radiation patterns, were measured. The top and bottom views of the prototype are shown in Figure 17a,b. Figure 17c,d shows the measurement setups for the S-parameter and 2D radiation pattern measurements.

**Figure 18:**
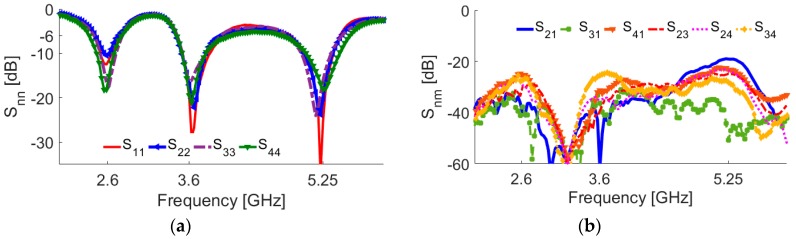
Measured (**a**) S_nn_ and (**b**) S_nm_ characteristics of the prototype.

The measurement results of the antenna S-parameters are illustrated in Figure 18. As illustrated, good S-parameters have been obtained for all the double-element slot radiators of the design. In agreement with the simulations, the prototype provides triple-band characteristics at 2.6, 3.6, 5.25 GHz resonant frequencies. In addition, high-isolation and low mutual-coupling properties have been achieved.

**Figure 19:**
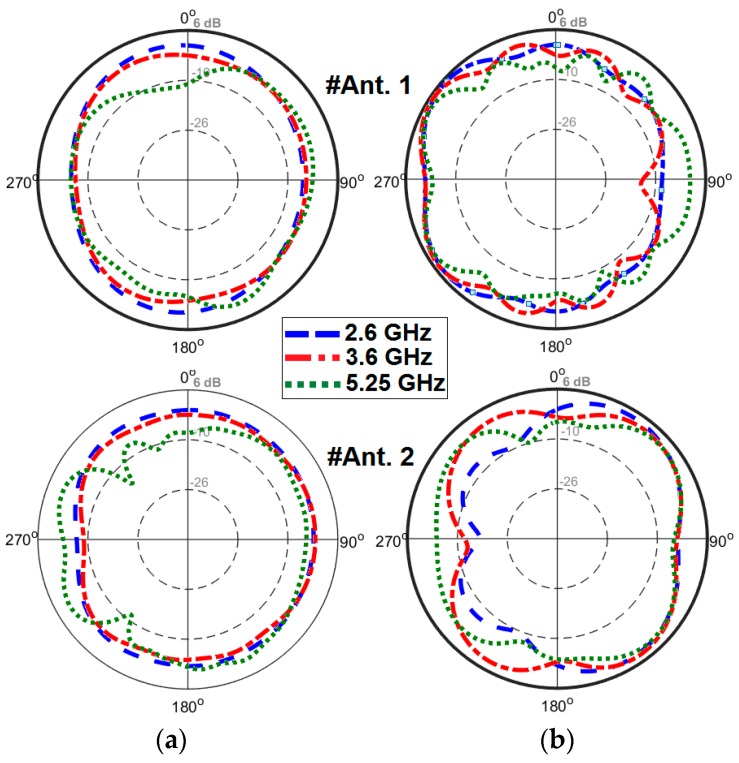
2D-polar radiation patterns in the (**a**) H-plane and (**b**) E-plane.

**Figure 20:**
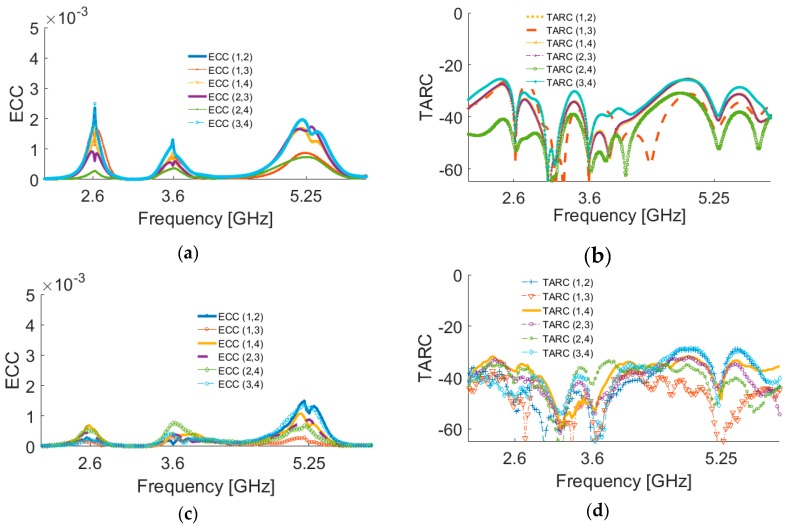
Simulated (**a**) envelope correlation coefficient (ECC) and (**b**) total active reflection coefficient (TARC), and measured (**c**) ECC and (**d**) TARC results.

2D-polar radiation patterns of the antenna at different resonant frequencies are depicted in Figure 19. Figure 19a shows that omnidirectional radiation patterns are achieved in the H-plane, especially at 2.6 and 3.6 GHz. The E-plane radiation patterns of the antenna, as shown in Figure 19b, look like an 8-shaped dumbbell due to the nature of the slot resonators. The envelope correlation coefficient (ECC) is an important parameter of the MIMO antenna system, quantifying its multiple port performance. A lower ECC means more diversified patterns [23]. An acceptable standard for a desirable MIMO system is ECC < 0.5. Another important parameter which must be considered in these systems is the total active reflection coefficient (TARC), which is defined as the square root of the ratio of reflected power to incident power [24]. Figure 20 shows the ECC and TARC results from the simulations and measurements. The calculated ECC results indicate that ECC is within acceptable limits and is very low over the antenna operation bands. As illustrated, TARC is less than −40 at the resonant frequencies.

## 4. User-Effect and SAR Investigation

**Figure 21:**
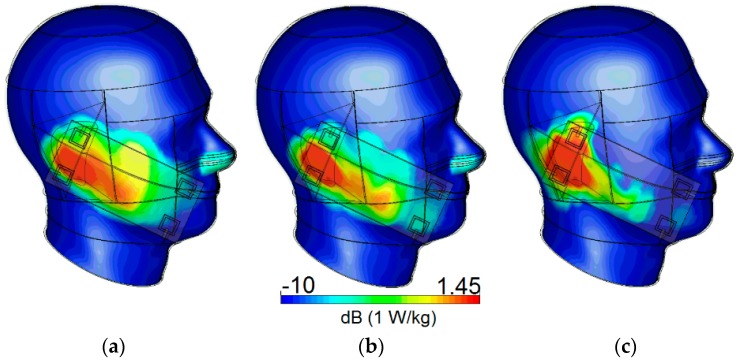
Simulated specific absorption rate (SAR) at (**a**) 2.6, (**b**) 3.6, and (**c**) 5.25 GHz.

In this section, the specific absorption rate (SAR) characteristic and the user-impact on the radiation characteristics of the antenna system are reported. SAR, one of the most critical issues of mobile terminal systems, is a measure of electromagnetic radiation absorbed by a human body, and should be as low as possible [25,26]. Figure 21 illustrates the simulated SAR characteristic in the vicinity of a human head for different resonant frequencies. The smartphone PCB has been placed with less than 10 mm distance of the user’s head.

**Figure 22:**
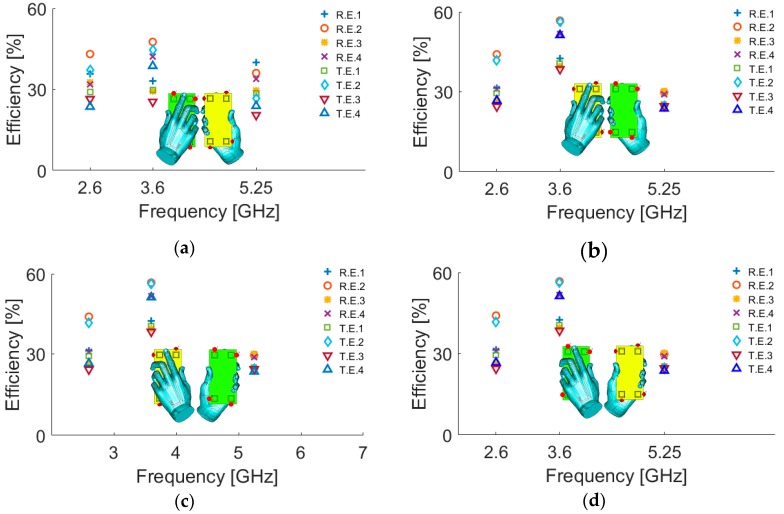
Efficiency characteristic of the antenna elements for different modes (**a**) RHM-1, (**b**) RHM-2, (**c**) LHM-1, and (**d**) LHM-2.

As shown, the antenna has low SAR values at 2.6, 3.6, and 5.25 GHz. The radiation efficiency and total efficiency of the antenna under different single hand modes are studied in Figure 22. The hand modes include right-hand mode on the top of PCB (RHM-1), right hand mode on the bottom of PCB (RHM-2), left hand mode on the top of PCB (LHM-1), and left hand mode on the bottom of PCB (LHM-2). According to the simulations, the antenna elements show good performances in the vicinity of the user’s hand and provide good radiation and total efficiencies. The maximum reduction in the antenna efficiency usually occurs when the antenna element is partially covered by hand tissue.

**Figure 23:**
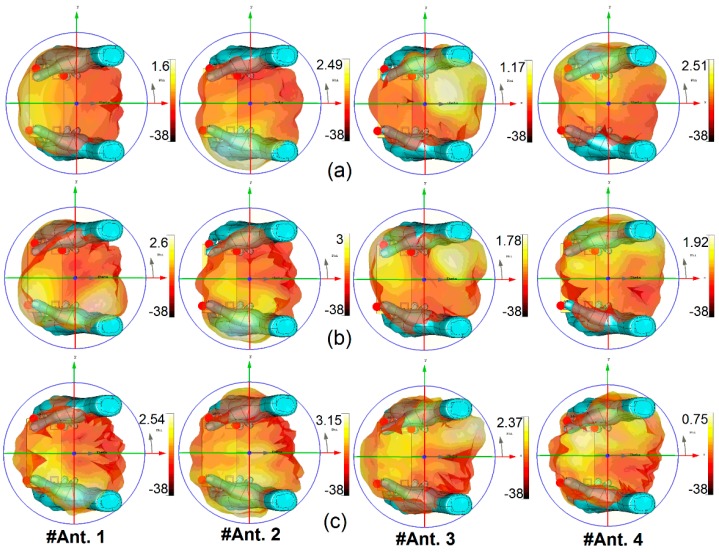
3D Radiation patterns of the antenna elements in the present of both user’s hands at (**a**) 2.6 GHz, (**b**) 3.6 GHz, and (**c**) 5.25 GHz.

3D radiation patterns of the antenna elements at the resonant frequencies (2.6, 3.6, and 5.25 GHz) when held by both hands are shown in Figure 23. Generally, a user’s hand reduces the radiation performance of the antenna, but for the proposed antenna, as illustrated, the radiation patterns retain sufficient gain and pattern coverage. The minimum gain value is obtained for #Ant. 4 at 5.25 GHz. The antenna elements provide different gain values, varying from 0.75 dB to 3.15 dB IEEE gain.

**Figure 24:**
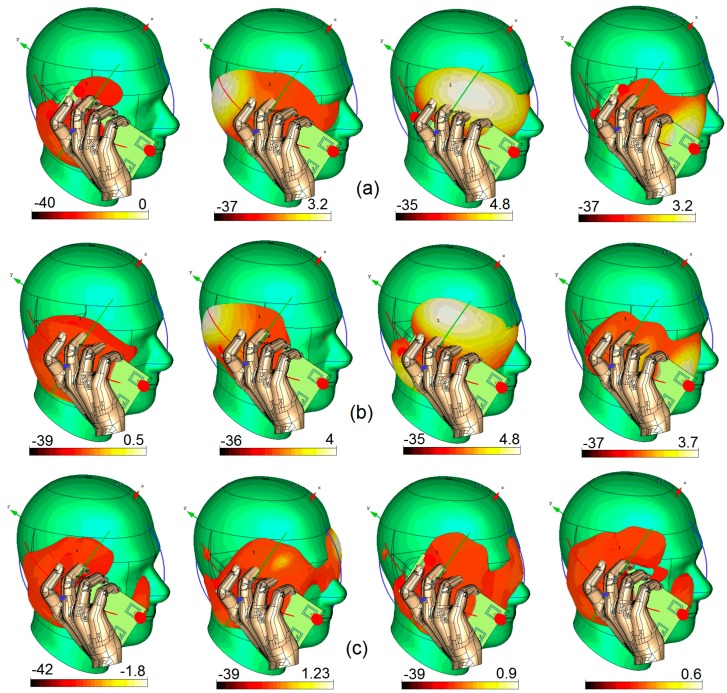
3D Radiation pattern results in Talk-Mode at (**a**) 2.6 GHz, (**b**) 3.6 GHz, and (**c**) 5.25 GHz.

Figure 24 illustrates the radiation patterns for each element of the antenna system in Talk-Mode: the antenna system works well and provides sufficient gain values for each radiator used at different corners of the PCB. However, in a few cases, the antenna elements provide low gain mainly because those antenna elements are covered by a hand and also are close to the head. For example, compared with the other radiators, #Ant.1 cannot provide sufficient gain at various frequencies. For the other radiators, the obtained IEEE gain varies from 0.6 to 4.8 dB at different frequencies and mainly depends on the locations of the antenna element in the Talk-Mode scenario.

Table 2 compares the fundamental properties of the proposed MIMO antenna with MIMO antenna arrays recently reported in the literature [5,6,7,8,9,10,11,12,13,14,15]. As can be seen, the proposed antenna can support three different operation bands with very similar radiation and MIMO performances in terms of bandwidth, efficiency, isolation, and ECC. In contrast to the reported designs, each radiator of our design can cover 4G and 5G frequency operation bands simultaneously, a unique feature that none of the other designs cited have.

## 5. Conclusions

A multi-band MIMO antenna for future smartphone applications covering three spectral bands has been presented. To achieve the triple-band function, double-element square-ring slot radiators with the microstrip-line feeding technique are deployed at the corners of the smartphone PCB. The impedance-bandwidth of each radiator spans from 2.6 to 2.7 GHz, 3.45 to 3.8 GHz. and 5.0 to 5.45 GHz covering the 2.6, 3.6, and 5.25 GHz resonant frequencies, respectively. The antenna is a candidate for 4G and 5G applications. SAR and performance variation of the antenna with user hand and head interaction are made clear.

**Figure 25:**
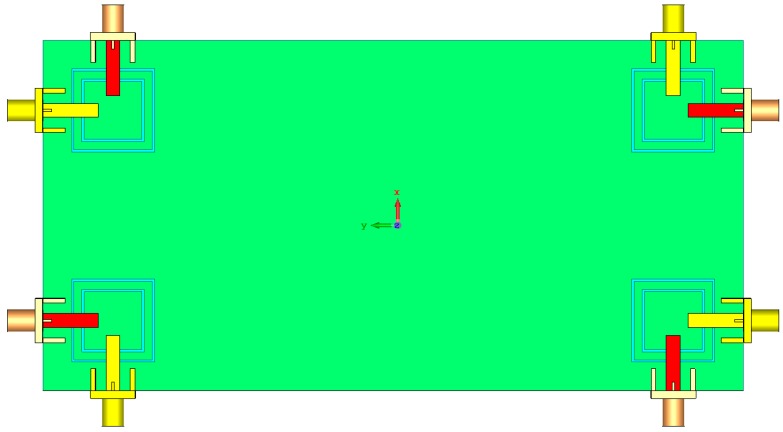
The new configuration of the proposed MIMO smartphone antenna design.

Due to the symmetrical configuration of the employed double-element slot resonators, there is a possibility to use them as dual-polarized resonators and increase the radiation elements of the proposed MIMO design by embedding more microstrip feed lines, as shown in Figure 25. Investigation of the resulting mutual coupling of adjacent elements must be investigated, a suitable topic for further work.

## Figures and Tables

**Table 1:** Parameter values of the designs.

Parameter	Value (mm)	Parameter	Value (mm)	Parameter	Value (mm)
W_Sub_	75	L_sub_	150	d	6
W_S_	15	L_f_	11.85	W_f_	3
g	0.5	W	17.8	W_1_	14
W_2_	18	W_3_	12.6	L_f2_	11.4

**Table 2:** Comparison between the presented and reported mobile handset antennas.

Reference	Bandwidth (GHz)	Efficiency (%)	Overall Size (mm^2^)	Isolation (dB)	ECC
[5]	2.55–2.68	48–63	136 × 68	12	>0.15
[6]	3.4–2.3.8 5.15–5.92	41–84 47–79	150 × 80	12	>0.15
[7]	2.55–2.6	48–63	136 × 68	11	>0.15
[8]	3.4–3.6	62–78	140 × 70	10	>0.20
[9]	3.4–3.8	55–70	150 × 75	15	<0.05
[10]	3.55–3.65	52–76	150 × 75	11	—
[11]	1.88–1.92 2.30–2.62	39–55 50–70	138 × 68.8	10	<0.15
[12]	0.63–0.96 1.70–2.70 3.50–3.80	40–60 50–75 60–75	130 × 70	—	—
[13]	0.82–0.96 1.8–2.6 3.4–3.6	30–45 30–80 50–70	140 × 70	10	<0.4
[14]	0.84–0.96 1.72–2.65 3.40–3.60	10–50 10–80 20–90	150 × 74	13	<0.05
[15]	0.63–0.96 1.70–2.70 3.50–3.80	35–48 10–75 55–87	40 × 65	10	<0.35
Proposed	2.5–2.7 3.45–3.8 5–5.45	64–75 73–76 69–75	150 × 75	17	<0.05
